# Biodegradation of Spilled Diesel Fuel in Agricultural Soil: Effect of Humates, Zeolite, and Bioaugmentation

**DOI:** 10.1155/2014/642427

**Published:** 2014-01-08

**Authors:** Pavel Kuráň, Josef Trögl, Jana Nováková, Věra Pilařová, Petra Dáňová, Jana Pavlorková, Josef Kozler, František Novák, Jan Popelka

**Affiliations:** ^1^Research Institute of Inorganic Chemistry, a.s., Revoluční 84, 400 01 Ústí nad Labem, Czech Republic; ^2^Jan Evangelista Purkyně University in Ústí nad Labem, Faculty of Environment, Králova Výšina 3132/7, 400 96 Ústí nad Labem, Czech Republic; ^3^MikroChem LKT, spol. s r.o. Přeseka 52, 379 01 Třeboň, Czech Republic; ^4^Biological Centre AV ČR, v.v.i, Institute of Soil Biology, Na Sádkách 7, 370 05 České Budějovice, Czech Republic

## Abstract

Possible enhancement of biodegradation of petroleum hydrocarbons in agricultural soil after tank truck accident (~5000 mg/kg dry soil initial concentration) by bioaugmentation of diesel degrading *Pseudomonas fluorescens* strain and addition of abiotic additives (humates, zeolite) was studied in a 9-month pot experiment. The biodegradation process was followed by means of analytical parameters (hydrocarbon index expressed as content of C_10_–C_40_ aliphatic hydrocarbons, ratio pristane/C_17_, and total organic carbon content) and characterization of soil microbial community (content of phospholipid fatty acids (PLFA) as an indicator of living microbial biomass, respiration, and dehydrogenase activity). The concentration of petroleum hydrocarbons (C_10_–C_40_) was successfully reduced by ~60% in all 15 experiment variants. The bioaugmentation resulted in faster hydrocarbon elimination. On the contrary, the addition of humates and zeolite caused only a negligible increase in the degradation rate. These factors, however, affected significantly the amount of PLFA. The humates caused significantly faster increase of the total PLFA suggesting improvement of the soil microenvironment. Zeolite caused significantly slower increase of the total PLFA; nevertheless it aided in homogenization of the soil. Comparison of microbial activities and total PLFA revealed that only a small fraction of autochthonous microbes took part in the biodegradation which confirms that bioaugmentation was the most important treatment.

## 1. Introduction

Aliphatic petroleum hydrocarbons are the most abundant environmental pollutants. However, due to their extreme nonpolarity with preferential binding to soil organic matter, their residual concentrations in soils are often high, despite fair biodegradability (as compared to other, more recalcitrant, organic pollutants) [1, 2]. Bioremediation is considered to be a cost-effective and environmental-friendly approach for decontamination of polluted soils [3–5]. Nevertheless, the natural biodegradation in polluted soils is often slow due to factors such as high hydrocarbons concentrations, joint pollution with other pollutants (heavy metals), limiting nutrient content, insufficient water or oxygen supply, or low bioavailability of pollutants [5–7]. Developed bioremediation methods for organically polluted soils follow two main strategies, often combined together, that is, bioaugmentation (inoculation of soil with allochtonic, independently cultivated degrading microorganisms) and biostimulation (support of autochthonic (indigenous) degrading microorganism by abiotic additives) [2, 6]. Biostimulation is carried out predominantly by improvement of the level of soil nutrients; nevertheless recent effort has been oriented into testing of other abiotic additives [7].

Humic substances were investigated previously as additives in bioremediation of polychlorinated biphenyls [8], aromatics [9–11], chlorinated aliphatics [12], and petroleum hydrocarbons [13]. Their clear role in bioremediation is not established yet, especially regarding generally less explored bioremediation of aliphatic hydrocarbons as compared to aromatic or halogenated pollutants. Humic substances possess many functional groups and have good sorption characteristics. Soils with higher humate content have higher retention capacity for water and nutrients, improved pH and redox buffering, or higher organic content, which all lead to improved soil fertility [14]. Both organic pollutants and heavy metals are well sorbed on humic substances [10, 14, 15]. From the bioremediation point of view this usually leads to immobilization and consequent decrease in pollutant toxicity [11, 12]. On the other hand, humic substances can increase bioavailability of pollutants for degrading microorganisms [8], among other, by acting as surfactants [9, 11].

The role of zeolites in the biodegradation was studied much less. Overall zeolites are considered to improve soil quality by increasing retention capacity for water and nutrients. Zeolites and other porous inorganic materials can also bind microorganisms and thus serve as a good carrier for bioaugmentation [12, 16]. Significant improvement of the biodegradation of pentachlorophenol was observed with humic substances bound on zeolite (the so-called organomineral complex) [12].

The aim of this study was to assess, in *ex situ *laboratory pot experiment, the effect of addition of humates and zeolite as well as bioaugmentation on the rate of biodegradation of spilled diesel (∼5000 mg/kg) in loamy agricultural soil after tank truck accident. The process was monitored via elimination of C_10_–C_40_ aliphatic hydrocarbons and total organic carbon (TOC) as required by European waste legislation (Council directive 1999/31/EC [17]). Soil microorganisms were monitored by determination of dehydrogenase activity and respiration, known to be indicative of proceeding biodegradation [13], and phospholipid fatty acid (PLFA) content, common method for estimation of living microbial biomass in soils [18, 19], sediments [20], and other matrices [21].

## 2. Materials and Methods

### 2.1. Soil

A loamy agriculture soil (ash content 7%, moisture 10%, pH 7.5, N : P∼6 : 1, and TOC 4.0% dry weight) was used. The soil was freshly polluted by diesel fuel (compliant with EN590 [22], temperate climatic zones) as a consequence of tank truck accident. The soil was removed from the site and ∼1000 kg was transported directly to laboratory. Significant portion of stones was eliminated by sieving through a 1 cm sieve.

### 2.2. Humates

Two types of humates from oxyhumolite (Duchcov, Czech Republic) and young brown coal (lignite, Mikulčice, Czech Republic [23]) were prepared by alkali extraction [24].

### 2.3. Zeolite

The zeolite of size 1.5–2 mm was purchased from Zeopol, a. s. (Brno, Czech republic), and added at concentration of 100 g/kg wet soil.

### 2.4. Cultivation of Augmented Bacteria

*Pseudomonas fluorescens *diesel degrading strain from the culture collection of MikroChem LKT was cultivated ∼one week in Bacterial Salt Medium (BSM) [25] with diesel oil (∼1%) serving as a sole source of carbon and energy to an early exponential phase (2.3 × 10^6^ ± 0.5 × 10^6^ CFU/mL) and augmented in a concentration of 1.7 ± 0.4 × 10^6^ CFU/g wet soil once in the beginning of the experiment.

### 2.5. Experiment Variants

The experiment consisted of a total of 270 pots (conical pots, bottom diameter 135 mm, upper diameter 180 mm, and height 155 mm) with conical underplate (bottom diameter 150 mm, upper diameter 185 mm, height 25 mm) containing ∼2.5 kg of wet soil each: 15 variants of the experiment with different composition (Table 1) were prepared into 18 pots each (designed for six triplicate sampling).

Firstly, 2 kg of soil was weighted in 10 L plastic pail. For variant I 180 mL of water was added to soil and the mixture was rigorously stirred by a mixing device with special attachment allowing perfect homogenization of obtained mixture. For variant II 100 mL of bacterial suspension and 80 mL of water were added to the soil and the mixture was rigorously stirred. For variant XV 100 mL of bacterial suspension was mixed with 200 g of zeolite and 80 mL of water was added to the soil; the mixture was rigorously stirred.

For variants III–VIII 45 g of aqueous humate solution of required concentration was divided into three portions and subsequently added to soil, the mixture was rigorously stirred after addition of every portion of humate solution, 100 mL of bacterial suspension was added to obtained mixture, the rest of humate and bacterial suspension in beakers was washed with 35 g of water and combined with soil, and the mixture was again rigorously stirred.

For variants IX–XIV 45 g of aqueous humate solution of required concentration was divided into three portions and subsequently added to the soil, the mixture was rigorously stirred after addition of every portion of humate solution, 100 mL of bacterial suspension combined with 200 g of zeolite was added to the obtained mixture, the rest of humate and inoculate in beakers was washed with 35 g of water and combined with soil, and the mixture was again rigorously stirred.

The resulting mixture (soil with or without additives) was transferred from 10 L plastic pail to experimental pot and packed down by plastic spoon to prevent formation of the preferential channels in soil. Finally, the filled pots were transported to laboratory designed for pot experiments and covered with filter papers to slow down the water evaporation.

The soil moisture was maintained at ∼12–15% throughout the experiment. Two times a week the moisture measurement with a soil moisture-meter PCE-SMM1 (PCE GROUP, Meschede, Germany) and subsequent soil irrigating with distilled water were carried out.

### 2.6. Sampling

Samples were withdrawn at days 0, 28, 91, 147, 203, and 273 after the experiment startup. For each variant three entire pots were discarded. Aliquots for determination of soil moisture in particular pots were stored at ambient temperature maximally 3 days prior to determination. Aliquots for determination of dehydrogenase activity and respiration were stored under refrigerating conditions (4°C) and determined one week after sampling. Aliquots for determination of PLFA were frozen (−30°C) immediately and analyzed later.

All other determinations (C_10_–C_40_, pH, TOC, elemental composition, and N : P ratio) were carried out from dried soil, which was sieved through a 3.15 mm sieve for these purposes.

### 2.7. Analyses

Aliphatic hydrocarbon content expressed as the total concentration of C_10_–C_40_ was determined according to optimized method for soils and sludge [26]. Briefly, the soil was firstly extracted with acetone (p. a., Lach-Ner, Neratovice, Czech Republic); then the solution of hexane (95+ Pestapur, for pesticide residual analysis, Chromservis, Prague, Czech Republic) containing alkanes C_10_ and C_40_ was added and the soil was further extracted by stirring on magnetic stirrer. Finally, the extract was purified by florisil (60–100 mesh, LGC Promochem, Chromservis, Prague, Czech Republic). The content of C_10_–C_40_ in purified extract was determined by means of gas chromatograph HP 6890 series II coupled with flame ionization detector (GC-FID) on the column Equity-5, 30 m × 0.25 mm × 0.25 *μ*m, with temperature program 50°C, 2 min, 20°C/min, 310°C, 18.5 min. The detector temperature was 310°C, and injector temperature was 300°C. The injected volume was 1 *μ*L with splitless 0.2 min. The linear velocity of nitrogen as carrier gas was 30 cm/s.

The ratios of pristane/C_17_ and phytane/C_18_ were determined from the chromatograms of C_10_–C_40_ determination on the basis of calibration curves for all four analytes.

The total organic carbon content (TOC) was determined according to ISO 14235 [27] used in agricultural laboratories of the Czech Republic.

Phospholipid fatty acids were analyzed by the adopted method of Zelles [18] using frozen soil samples. Briefly, the total soil lipids were extracted by a single-phase mixture of methanol, chloroform, and phosphate buffer (0.05 M, pH 7.4) in the ratio 2 : 1 : 0.8. Lipids were fractionated into nonpolar lipids, glycolipids, and polar lipids on polar silica columns (Supelclean LC-Si, Sigma-Aldrich). Polar lipid fraction was subjected to mild alkaline methanolysis and prepared fatty acid methylesters were analyzed by means of GC-MS. Total PLFAs were quantified using methyl nonadecanoate internal standard [21].

Basal soil respiration (without substrate induction or moisture adjustment) was measured according to an adopted method [28–30] in 100 mL glass bottles (“pyrex”) with ∼1 g of wet fresh soil. The evolved CO_2_ was captured in 0.5 M NaOH and determined by HCl (0.1 M) titration after BaCl_2_ addition. The activity was expressed in nmol of CO_2_ evolved per minute from 1 g of dry soil.

Dehydrogenase activity in soil was determined according to slightly modified standard method (ISO 23753-1 [31]). 2 g of fresh soil was incubated in 5 mL of solution of triphenyltetrazolium chloride (TTC, 1 g/L) in Tris buffer (12.1 g/L, pH 7.6) at 25°C. Then, the produced triphenylformazan (TPF) was determined by means of spectrophotometry (546 nm) after acetone extraction. Absorbance of the background (obtained by incubation of 5 g in Tris buffer without TPF) was subtracted. The activity was expressed in mU per gram of dry soil (i.e., nmol of evolved TPF per minute per gram of dry soil).

### 2.8. Calculations and Statistics

Time course of C_10_–C_40_ elimination was fitted by zero-order kinetics (1) and first-order kinetics (2) using QCExpert statistical pack (Trilobyte software, Czech Republic):

(1) $$\begin{array}{c} c=c_{0}-k_{0}t, \end{array}$$

(2) $$\begin{array}{c} c=c_{l}+\left(c_{0}-c_{l}\right)e^{-k_{1}t}, \end{array}$$

where *c* represents instantaneous concentration at time *t*, *c*_0_ is initial concentration, *c*_*l*_ is limit (asymptotical, “final”) concentration, *t* is time, and *k*_0_ and *k*_1_ are zero-order and first-order rate constants (regression parameters), respectively. Elimination half-lives were calculated as

(3) $$\begin{array}{c} t_{1/2}=\frac{k_{1}}{\ln2}. \end{array}$$

Mann-Whitney test and Bonferroni intervals were calculated using STATGRAPHICS Centurion XVI (StatPoint Technologies, USA), ANOVA, MANOVA, and Bonferroni tests for PFLA analysis using Statistica 10 (StatSoft, USA). All tests were evaluated at *α* = 0.05 significance level; all confidence intervals were calculated at 95% confidence level.

## 3. Results and Discussion

### 3.1. Aliphatic Hydrocarbons Biodegradation

**Figure 1:**
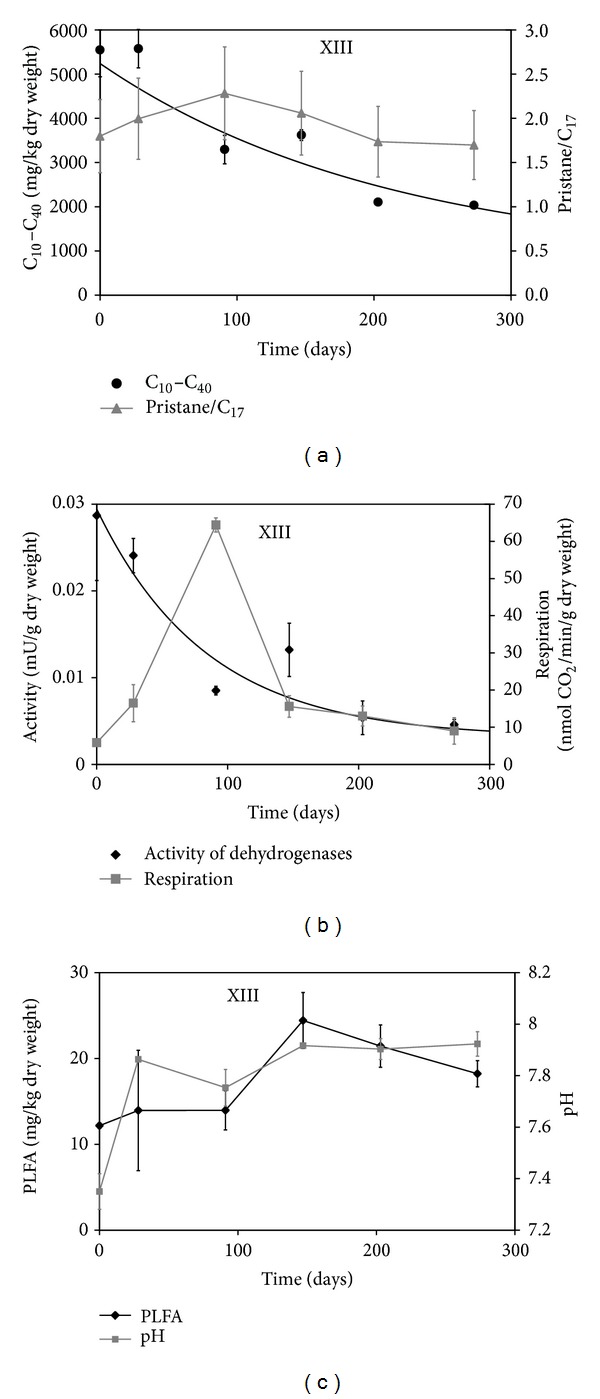
Typical time course of biodegradation process (variant XIII—bioaugmented soil with addition of zeolite and humate from oxyhumolite 150 mg/kg dry weight). Average values ± standard deviation are plotted. (a) Concentration of C_10_–C_40_ and pristane/C_17_ ratio (b) Soil respiration and dehydrogenase activity. (c) Soil PLFA and pH.

Typical course of C_10_–C_40_ biodegradation together with other important parameters is depicted in Figure 1 (individual charts for all variants are presented in Supplementary Material in Figure S1 (Supplementary Material available online at http://dx.doi.org/10.1155/2014/642427)).

Concentrations of C_10_–C_40_ aliphatic hydrocarbons decreased significantly from initial 5240 ± 440 to final 2330 ± 270 mg/kg dry soil; both values were comparable (ANOVA) for all variants. In four variants (III, IV, V, XI, all with lignite addition) significant decrease of C_10_–C_40_ concentration was detected already at day 28; other variants exhibited at least a 28-day lag and significant decrease was first detected at day 91.

Slight preferential biodegradation of n-alkanes as compared to branched alkanes was confirmed by increase of the pristane/C_17_ ratio [32]. Due to data variability, the increase was significant for a few individual variants only; however it was significant overall; see Table S2 in Supplementary Material. Biodegradation of both pristane and linear C_17_ followed the first-order kinetics (in the later samples concentrations of these indicator compounds were very close to the limits of quantification), but biodegradation of pristane was somewhat delayed. A very similar pattern was determined also for phytane/C_18_ ratio (not shown). Decrease of characteristic homologous series of paraffin (n-alkanes) sticking out from the envelope of nonseparated diesel oil complex mixture at the beginning of experiment in the whole range (C_10_–C_40_ n-alkanes) could be read from chromatograms (see Figure S2 in Supplementary Material). These also show the preferential biodegradation of short-chained alkanes (C_10_–C_25_) in comparison to higher nonpolar hydrocarbons. No shifts of retention time maxima were observed. This pattern was typical for all variants.

**Figure 2:**
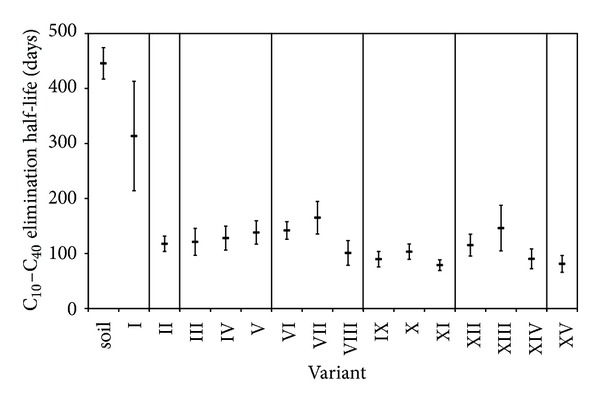
Comparison of half-lives of petroleum hydrocarbons (C_10_–C_40_) decrease (average values ±95%—confidence) in all variants. The variant “soil” stands for stored soil from the locality without any treatment. *R*-squared ranged from 0.83 to 0.97.

Half-lives of C_10_–C_40_ elimination are depicted in Figure 2. Compared to nonaugmented control (variant I, the slowest biodegradation), the half-lives of C_10_–C_40_ elimination in other variants were significantly shorter. The shortest half-lives were observed for the combination of all three factors (bioaugmentation + zeolite + humate 450 mg/g, variants XI and XIV) and for combination of zeolite and bioaugmentation (variant XV). This suggests a positive effect of zeolite addition and a synergic effect of the treatments. Nevertheless this conclusion remains in the hypothesis level, since due to data variability even these shortest half-lives were mutually comparable to half-lives of other bioaugmented variants. All variants exhibited significantly shorter half-lives as compared to soil from the locality, which was not treated in any way. This result confirms the importance of irrigation and aeration during bioremediation of petroleum hydrocarbons [2, 6].

In accordance with the biodegradation progress TOC concentration decreased significantly in all variants from initial 3.8 ± 0.2% to final 3.1 ± 0.1% (details are presented in Supplementary Material as Table S1). Final TOC concentrations were comparable for all variants.

Soil pH increased significantly during the experiment period from initial 7.46 ± 0.14 to final 7.98 ± 0.09 (Table S3 in Supplementary Material). Initial pH values were increased for variants with humates addition. Final pH values were significantly higher for augmented variants; other joint factors did not affect the final pH values.

### 3.2. Soil Microbial Communities during Biodegradation

The presence of microbial strains capable of degrading pollutants under given conditions is a prerequisite for successful bioremediation. Soil respiration exhibited a peak-type course [33] with initial respiration comparable to zero and maximum respiration at day 91 (Figure 1(b)) corresponding to maximum biodegradation of aliphatic hydrocarbons. Using Mann-Whitney test (used due to the absence of normal distribution of the respiration data) insignificant differences between individual variants were revealed; nevertheless, when evaluated jointly, the variants with humates without zeolite (III to VIII) exhibited significantly higher respiration than others. Dehydrogenase activities exhibited a first-order decrease in accordance with the decrease of hydrocarbons content (Figure 1(b)). The differences between variants were insignificant, but when evaluated jointly the variants with zeolite addition (IX to XV) exhibited significantly lower dehydrogenase activities compared to bioaugmented variants without zeolite (II to VIII).

The concentrations of soil PLFA in all variants increased during initial 147 days and then stagnated or slowly decreased (Figure 1(c)). Increase in the soil PLFA during the first half of the experiment indicates usual proliferation of soil microorganisms during biodegradation [13, 34, 35] and it is in accordance with increasing respiration rate and hydrocarbon elimination. Evaluated individually, differences in PLFA concentrations between variants were insignificant. Considering humates (regardless of concentration and humate type) and zeolite as joint factors (Table 2), significant changes could be observed in the midcourse of the experiment (days 91 to 203). Addition of zeolites resulted in significantly lower PLFA concentrations while addition of humates resulted in significantly higher PLFA concentrations (MANOVA and Bonferroni test).

Absolute concentrations of PLFA in used polluted soil (8.9 to 24.0 mg/kg dry mass) are comparable to similar nonpolluted meadows or arable agricultural soils with intensive management [36]. On the other hand, the soil respiration (0 to 60 nmol/min/g dry soil) is comparable rather to poorer soils such as younger phases of primary succession [30] or deep subsurface soil [28]. Decrease in microbial activity is even more pronounced if we count in the initial optimization of moisture content as well as higher temperature (25°C) during respiration measurement as compared to more usual 20°C [28, 30, 37, 38]. This comparison suggests that the diesel spill affected negligibly the viability of soil microorganisms but it rather inhibited their metabolic activities, that is, it shifted them to an inactive state.

### 3.3. Bioaugmentation Was the Most Significant Treatment

Bioremediation is a complex process with many potentially influencing factors, often unclear [33]. Deeper discussion of obtained data is therefore needed in order to draw conclusions. The C_10_–C_40_ data indicate low effect of addition of humates (variants III–VIII), zeolite (variant XV), or their combination (variants IX–XIV) on the rate of hydrocarbons biodegradation (biodegradation half-lives, Figure 2) upon bioaugmentation. Despite that the data indicate a possible positive effect of both additives, the increase in biodegradation rate was insignificant and obtained half-lives were comparable to variant II (bioaugmented soil only). On the contrary, the bioaugmentation of *P. fluorescens *degrading strain increased the biodegradation rate (significantly lower half-lives of all bioaugmented variants II to XV as compared to nonaugmented variant I; Figure 2). This observation is even supported by comparison of kinetic models (zero-order versus first-order kinetics). Based on the calculation of Akaike criterion [39], for bioaugmented variants (II to XV) the C_10_–C_40_ concentration decrease was better explained by first-order kinetics (2). On the contrary, the nonaugmented control (variant I) was better explained by the zero-order kinetics (1) indicating limitation by insufficient catalyst (i.e., degrading microorganisms). In accordance with the low microbial activity discussed in the previous section these data suggest insufficient number of degrading microorganisms in the original soil. Thus the bioaugmentation of degrading *P. fluorescens *strain was the most important treatment in the effort to increase the rate of biodegradation.

### 3.4. Effects of Humates and Zeolite on the Bioremediation Process

Opposite to expectation, no clear and significant effect of addition of humates or zeolite on the rate of biodegradation was found. Possible synergic effect of combination of humates and zeolite was indicated, but not significant. In addition, these additions had no effect on the terminal C_10_–C_40_ concentrations which were comparable in all variants. A few hypotheses can be formulated to explain why positive effects were not more distinct. First, our experiment integrated several established bioremediation techniques—that is, bioaugmentation of degrading strain and biostimulation by optimization of moisture content. Both techniques are known to be crucial for successful bioremediation [34] and could overwhelm the positive effects of humates and zeolite. Indeed, several studies demonstrated biostimulation to be a more important treatment than bioaugmentation [6, 13, 34]. Among others humic substances are considered to serve as surfactants [14] increasing pollutant bioavailability. The pollution in our case was fresh (i.e., more bioavailable [40]) which could blunt the positive effect of humates.

On the contrary a clear positive effect of humates on soil microbial community (increased PLFA, increased respiration rate) was observed in accordance with the study of Turgay et al. [13]. Viable and active soil microbial community is an important attribute of fertile and functional soil [14]. This is not, however, the case of polluted soils, where microbial communities are often disrupted [19] and which was also the case of our used soil. Microbial data suggested that despite the negligible effect on the biodegradation the addition of humates was not purposeless and it can contribute to the success of the bioremediation process by aiding in the restoration of microbial community.

In case of zeolite, it is likely that both augmented bacteria and indigenous soil bacteria were adsorbed on it [16] thus causing the observed decrease of PLFA and dehydrogenase activity. The temporal character of this decrease indicates that in longer-term scale also zeolite addition might be useful. At last it should be also noted that the presence of zeolite in the soil enabled simple visual monitoring of soil homogenization.

## 4. Conclusion

Successful biodegradation of fresh diesel fuel spill in loamy agricultural soil was achieved. While bioaugmentation was revealed to be the most important factor for increase of the biodegradation rate, the effect of humates and zeolite was only negligible. On the other hand the humates had positive effect on soil microbial community which may be of importance for restoration of soil function and fertility.

## Supplementary Material

The supplementary material contains more detailed information about the biodegradation process, i.e. course of biodegradation in all experiment variants; details on TOC, pristane/C17, and pH including statistical evaluation and sample GC-FID chromatograms of C10-C40 aliphatic hydrocarbons showing preferential biodegradation of n-alkanes and shorter-chained alkanes.

## Figures and Tables

**Table 1:** Experiment variants.

Variant	Composition
I	Soil
II	Soil + bacteria
III	Soil + bacteria + HS from lignite 50 mg/kg dry weight
IV	Soil + bacteria + HS from lignite 150 mg/kg dry weight
V	Soil + bacteria + HS from lignite 450 mg/kg dry weight
VI	Soil + bacteria + HS from oxyhumolite 50 mg/kg dry weight
VII	Soil + bacteria + HS from oxyhumolite 150 mg/kg dry weight
VIII	Soil + bacteria + HS from oxyhumolite 450 mg/kg dry weight
IX	Soil + bacteria + zeolite + HS from lignite 50 mg/kg dry weight
X	Soil + bacteria + zeolite + HS from lignite 150 mg/kg dry weight
XI	Soil + bacteria + zeolite + HS from lignite 450 mg/kg dry weight
XII	Soil + bacteria + zeolite + HS from oxyhumolite 50 mg/kg dry weight
XIII	Soil + bacteria + zeolite + HS from oxyhumolite 150 mg/kg dry weight
XIV	Soil + bacteria + zeolite + HS from oxyhumolite 450 mg/kg dry weight
XV	Soil + bacteria + zeolite

abbr.: HS: humate.

**Table 2:** Total phospholipid fatty acids (mg PLFA/kg dry soil, average values ±95% Bonferroni intervals) as an effect of additives in bioaugmented variants (II to XV).

Time (days)	Zeolite (XV)	Humates + zeolite (IX–XIV)	Humates only (III–VIII)	No amendments (II)
0	8.9 ± 3.4^abc^	12.7 ± 1.6^a^	13.6 ± 2.3^ab^	15.7 ± 7.5^abc^
28	11.6 ± 2.8^abc^	12.4 ± 1.7^a^	16.1 ± 2.4^abcd^	15.0 ± 13.0^abcd^
**91**	16.5 ± 3.4^**abcdef**^	13.3 ± 1.5^**a**^	21.7 ± 2.2^**ef**^	12.0 ± 7.5^**abcde**^
**147**	16.3 ± 2.8^**abcdef**^	18.7 ± 1.6^**cde**^	24.0 ± 2.2^**f**^	16.6 ± 7.5^**abcde**^
203	21.1 ± 2.8^abcdef^	21.1 ± 1.7^*def*^	22.6 ± 2.0^ef^	19.4 ± 7.5^abcdef^
273	15.2 ± 3.4^abcdef^	18.3 ± 1.5^bcde^	20.7 ± 2.0^*def*^	22.7 ± 13.0^abcdef^

Letters indicate homogenous groups of the values (Bonferroni test).

Bold-font rows indicate sampling times with significant differences in PLFA concentrations.
