# Supplementary material for: Biodegradation of Spilled Diesel Fuel in Agricultural Soil: Effect of Humates, Zeolite, and Bioaugmentation
Source: ScientificWorldJournal. 2014 Jan 8;2014:642427. doi: 10.1155/2014/642427 (PMC3930029; doi:10.1155/2014/642427)
Supplement: Supplementary file 1 — The supplementary material contains more detailed information about the biodegradation process, i.e. course of biodegradation in all experiment variants; details on TOC, pristane/C17, and pH including statistical evaluation and sample GC-FID chromatograms of C10-C40 aliphatic hydrocarbons showing preferential biodegradation of n-alkanes and shorter-chained alkanes. [file 642427.f1.doc]

I – soil a b c

;

II – soil + bacteria a b c

III – Soil + bacteria + HS from lignite 50 mg/kg dry weight

a b c

IV – Soil + bacteria + HS from lignite 150 mg/kg dry weight

a b c

V – Soil + bacteria + HS from lignite 450 mg/kg dry weight

a b c

VI – Soil + bacteria + HS from oxyhumolite 50 mg/kg dry weight

a b c

VII – Soil + bacteria + HS from oxyhumolite 150 mg/kg dry weight

a b c

VIII – Soil + bacteria + HS from oxyhumolite 450 mg/kg dry weight

a b c

IX – Soil + bacteria + zeolite + HS from lignite 50 mg/kg dry weight

a b c

X – Soil + bacteria + zeolite + HS from lignite 150 mg/kg dry weight

a b c

XI – Soil + bacteria + zeolite + HS from lignite 450 mg/kg dry weight

a b c

XII – Soil + bacteria + zeolite + HS from oxyhumolite 50 mg/kg dry weight

a b c

XIII – Soil + bacteria + zeolite + HS from oxyhumolite 150 mg/kg dry weight

a b c

XIV – Soil + bacteria + zeolite + HS from oxyhumolite 450 mg/kg dry weight

a b c

XV – Soil + bacteria + zeolite a b c

Figure S1 Kinetics of indicators of biodegradation in individual variants. a) Concentration of aliphatic hydrocarbons (C_10_-C_40_) and ratio Pristane/C_17_ b) Microbial activities (activity of dehydrogenases and respiration) c) Total phospholipid fatty acids (PLFA) and pH. Concentration of C_10_-C_40_ and activity of dehydrogenases are fitted by first-order kinetics (equation 2). Plotted are average values ± standard deviations. HS = humates.


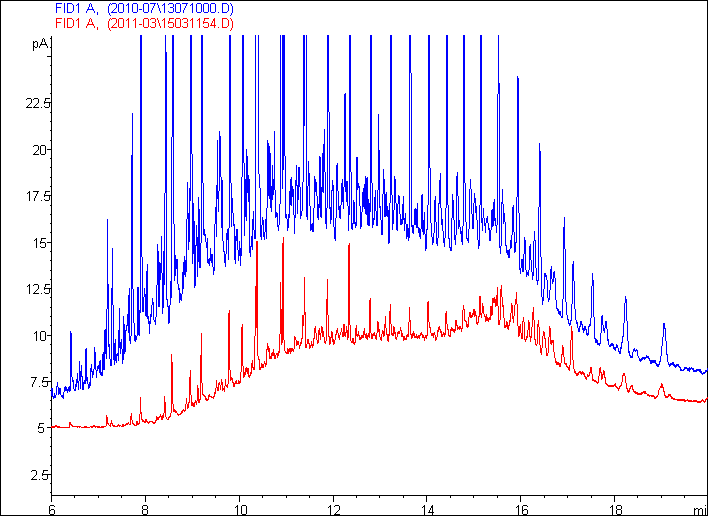


C_10_-C_25_

n-alkanes

C_17_, Pristane

C_18_, Phytane

Figure S2 Sample GC-FID chromatograms of C_10_–C_40_ content at the beginning (day 0, blue curves) and the end (day 273, red) of experiment in the same scale (variant V, soil + bacteria + humate from lignite 450 mg/kg dry weight). The peaks of characteristic homologous series of n-alkanes are sticking out from the envelope of non-separated crude oil complex mixture.

Table S1 Changes of total organic carbon (TOC) during the experiment. Shown are average values ± 95% Bonferroni confidences in independent comparisons for: a) individual variants, b) factors joining additives, c) comparison of inoculated and non-inoculated variants); letters indicate homogenous groups of the values (Bonferroni test); bold lines indicate significant changes between day 0 and day 273.

| **Factor** | **day 0** | | | | **day 273** | | |
| --- | --- | --- | --- | --- | --- | --- | --- |
| **a) Individual variants** | | | | | | | |
| **I** | | **3.98** | **±** | **0,21ab** | **3.02** | **±** | **0,21e** |
| **II** | | **3.85** | **±** | **0,21abc** | **3.13** | **±** | **0,21e** |
| **III** | | **3.92** | **±** | **0,21abc** | **3.04** | **±** | **0,21e** |
| **IV** | | **3.82** | **±** | **0,21abc** | **3.15** | **±** | **0,21e** |
| **V** | | **3.91** | **±** | **0,21abc** | **3.14** | **±** | **0,21e** |
| **VI** | | **4.06** | **±** | **0,21a** | **3.19** | **±** | **0,21e** |
| **VII** | | **3.87** | **±** | **0,21abc** | **3.09** | **±** | **0,21e** |
| **VIII** | | **3.85** | **±** | **0,21abc** | **3.27** | **±** | **0,21de** |
| **IX** | | **3.69** | **±** | **0,21bc** | **3.01** | **±** | **0,21e** |
| **X** | | **3.58** | **±** | **0,21cd** | **2.97** | **±** | **0,21e** |
| **XI** | | **3.71** | **±** | **0,21abc** | **3.10** | **±** | **0,21e** |
| **XII** | | **3.60** | **±** | **0,21cd** | **3.09** | **±** | **0,21e** |
| **XIII** | | **3.71** | **±** | **0,21abc** | **3.00** | **±** | **0,21e** |
| **XIV** | | **3.70** | **±** | **0,21abc** | **3.08** | **±** | **0,21e** |
| **XV** | | **3.78** | **±** | **0,21abc** | **3.12** | **±** | **0,21e** |
|  | |  |  |  |  |  |  |
| **b) Joined factors** | | | | | | | |
| **zeolite (XV)** | **3.78** | | **±** | **0,18ab** | **3.12** | **±** | **0,18c** |
| **humates+zeolite (IX-XIV)** | **3.67** | | **±** | **0,07a** | **3.04** | **±** | **0,07c** |
| **humates only (III-VIII)** | **3.91** | | **±** | **0,07b** | **3.15** | **±** | **0,07c** |
| **none (I+II)** | **3.92** | | **±** | **0,13b** | **3.07** | **±** | **0,13c** |
|  |  | |  |  |  |  |  |
| **c) Effect of inoculation** | | | | | | | |
| **Inoculated (II-XV)** | **3.79** | | **±** | **0,05a** | **3.09** | **±** | **0,05b** |
| **Non-inoculated (I)** | **3.98** | | **±** | **0,20a** | **3.01** | **±** | **0,20b** |

Table S2 Changes of ratio pristane/C_17_ during the experiment. Shown are average values ± 95% Bonferroni confidences in independent comparisons for: a) individual variants, b) factors joining additives, c) comparison of inoculated and non-inoculated variants); bold lines indicate significant changes between day 0 and day 273.

| **Factor** | **day 0** | | | **day 273** | | |
| --- | --- | --- | --- | --- | --- | --- |
| **a) Individual variants** | | | | | | |
| I | 1.63 | ± | 0,29a | 2.02 | ± | 0,29abcde |
| **qII** | **1.63** | **±** | **0,29a** | **2.53** | **±** | **0,29e** |
| III | 1.76 | ± | 0,29abc | 1.98 | ± | 0,29abcd |
| IV | 1.75 | ± | 0,29abc | 2.11 | ± | 0,29abcde |
| V | 1.66 | ± | 0,29ab | 2.06 | ± | 0,29abcde |
| VI | 1.68 | ± | 0,29ab | 2.08 | ± | 0,29abcde |
| **VII** | **1.73** | **±** | **0,29abc** | **2.32** | **±** | **0,29de** |
| **VIII** | **1.68** | **±** | **0,29ab** | **2.20** | **±** | **0,29cde** |
| IX | 1.77 | ± | 0,29abc | 1.79 | ± | 0,29abc |
| X | 1.80 | ± | 0,29abc | 1.79 | ± | 0,29abc |
| XI | 1.77 | ± | 0,29abc | 2.15 | ± | 0,29bcde |
| XII | 1.81 | ± | 0,29abc | 2.00 | ± | 0,29abcd |
| XIII | 1.80 | ± | 0,29abc | 1.70 | ± | 0,29abc |
| XIV | 1.85 | ± | 0,29abcd | 1.95 | ± | 0,29abcd |
| XV | 1.79 | ± | 0,29abc | 1.70 | ± | 0,29abc |
|  |  |  |  |  |  |  |
| **b) Joined factors** | | | | | | |
| zeolite (XV) | 1.79 | ± | 0,28abc | 1.70 | ± | 0,28ab |
| humates+zeolite (IX-XIV) | 1.80 | ± | 0,11ab | 1.90 | ± | 0,11b |
| **humates only (III-VIII)** | **1.71** | **±** | **0,11a** | **2.12** | **±** | **0,11cd** |
| **none (I+II)** | **1.63** | **±** | **0,20a** | **2.28** | **±** | **0,2d** |
|  |  |  |  |  |  |  |
| **c) Effect of inoculation** | | | | | | |
| **Inoculated (II-XV)** | **1.75** | **±** | **0,08a** | **2.02** | **±** | **0,08b** |
| Non-inoculated (I) | 1.63 | ± | 0,30a | 2.02 | ± | 0,3ab |

Table S3 Changes of pH during the experiment. Shown are average values ± 95% Bonferroni confidences in independent comparisons for: a) individual variants, b) factors joining additives, c) comparison of inoculated and non-inoculated variants); bold lines indicate significant changes between day 0 and day 273.

| **Factor** | **day 0** | | | **day 273** | | |
| --- | --- | --- | --- | --- | --- | --- |
| **a) Individual variants** | | | | | | |
| **I** | **7.46** | **±** | **0,14abcde** | **7.77** | **±** | **0,14fgh** |
| **II** | **7.26** | **±** | **0,14a** | **7.98** | **±** | **0,14hi** |
| **III** | **7.47** | **±** | **0,14abcde** | **7.96** | **±** | **0,14hi** |
| **IV** | **7.62** | **±** | **0,14def** | **7.98** | **±** | **0,14hi** |
| **V** | **7.69** | **±** | **0,14efg** | **7.96** | **±** | **0,14hi** |
| **VI** | **7.48** | **±** | **0,14abcde** | **7.93** | **±** | **0,14hi** |
| **VII** | **7.57** | **±** | **0,14cdef** | **7.99** | **±** | **0,14hi** |
| **VIII** | **7.42** | **±** | **0,14abcd** | **7.92** | **±** | **0,14ghi** |
| **IX** | **7.37** | **±** | **0,14abc** | **8.10** | **±** | **0,14i** |
| **X** | **7.31** | **±** | **0,14ab** | **8.00** | **±** | **0,14hi** |
| **XI** | **7.42** | **±** | **0,14abcd** | **8.04** | **±** | **0,14i** |
| **XII** | **7.55** | **±** | **0,14bcdef** | **8.11** | **±** | **0,14i** |
| **XIII** | **7.35** | **±** | **0,14abc** | **7.92** | **±** | **0,14ghi** |
| **XIV** | **7.43** | **±** | **0,14abcd** | **7.95** | **±** | **0,14hi** |
| **XV** | **7.49** | **±** | **0,14abcde** | **8.03** | **±** | **0,14i** |
|  |  |  |  |  |  |  |
| **b) Joined factors** | | | | | | |
| **zeolite (XV)** | **7.49** | **±** | **0,16ab** | **8.03** | **±** | **0,16c** |
| **humates+zeolite (IX-XIV)** | **7.40** | **±** | **0,07b** | **8.02** | **±** | **0,07c** |
| **humates only (III-VIII)** | **7.54** | **±** | **0,07a** | **7.95** | **±** | **0,07c** |
| **none (I+II)** | **7.35** | **±** | **0,11b** | **7.87** | **±** | **0,11c** |
|  |  |  |  |  |  |  |
| **c) Effect of inoculation** | | | | | | |
| **Inoculated (II-XV)** | **7.45** | **±** | **0,04a** | **7.99** | **±** | **0,04c** |
| **Non-inoculated (I)** | **7.46** | **±** | **0,16a** | **7.77** | **±** | **0,16b** |
